# The most remarkable migrants—systematic analysis of the Western European insect flyway at a Pyrenean mountain pass

**DOI:** 10.1098/rspb.2023.2831

**Published:** 2024-06-12

**Authors:** Will L. Hawkes, Toby Doyle, Richard Massy, Scarlett T. Weston, Kelsey Davies, Elliott Cornelius, Connor Collier, Jason W. Chapman, Don R. Reynolds, Karl R. Wotton

**Affiliations:** ^1^ Centre for Ecology and Conservation, University of Exeter, Cornwall Campus, Penryn, Cornwall TR10 9FE, UK; ^2^ Environment and Sustainability Institute, University of Exeter, Cornwall Campus, Penryn, Cornwall TR10 9FE, UK; ^3^ Swiss Ornithological Institute, Seerose 1, Sempach, 6204, Switzerland; ^4^ Department of Entomology, Nanjing Agricultural University, Nanjing, 210095, People's Republic of China; ^5^ Natural Resources Institute, University of Greenwich, Chatham, Kent SE10 9LS, UK; ^6^ Rothamsted Research, Harpenden, Hertfordshire AL5 2JQ, UK

**Keywords:** Pyrenees, insect migration flyway, monitoring, migration rates, movement ecology

## Abstract

In autumn 1950 David and Elizabeth Lack chanced upon a huge migration of insects and birds flying through the Pyrenean Pass of Bujaruelo, from France into Spain, later describing the spectacle as combining both grandeur and novelty. The intervening years have seen many changes to land use and climate, posing the question as to the current status of this migratory phenomenon. In addition, a lack of quantitative data has prevented insights into the ecological impact of this mass insect migration and the factors that may influence it. To address this, we revisited the site in autumn over a 4 year period and systematically monitored abundance and species composition of diurnal insect migrants. We estimate an annual mean of 17.1 million day-flying insect migrants from five orders (Diptera, Hymenoptera, Hemiptera, Lepidoptera and Odonata) moving south, with observations of southward ‘mass migration’ events associated with warmer temperatures, the presence of a headwind, sunlight, low windspeed and low rainfall. Diptera dominated the migratory assemblage, and annual numbers varied by more than fourfold. Numbers at this single site hint at the likely billions of insects crossing the entire Pyrenean mountain range each year, and we highlight the importance of this route for seasonal insect migrants.

## 1. Introduction

Insects are the most numerous terrestrial migrants, far surpassing terrestrial and aerial vertebrate migrations in terms of biomass and abundance [1,2]. Insects migrate to exploit seasonal resources, increase reproductive output, and/or evade habitat deterioration due to temperature changes, variations in food quality or disease risk, or to seek suitable sites to overwinter [1,3]. The life histories of North Temperate Zone migrants are often associated with a high reproductive advantage such that autumn migrants considerably outnumber spring influxes to northern latitudes, for example by an average of over twofold for hoverflies and over threefold for the silver Y moth (*Autographa gamma*) [4,5].

Insect migration studies are often limited to specific taxa, for example butterflies and moths, dragonflies or hoverflies [6–8], while whole assemblage studies are relatively rare. This is because small insect migrants are often invisible in a large landscape, necessitating specialist equipment such as entomological radars [9,10] or balloon-supported nets for their detection [2,11,12]. However, in some rare locations known as migration bottlenecks, aerial densities are greatly increased by topographical conditions, allowing the whole migratory assemblage to be studied at ground level. Relatively few insect migration bottlenecks are known, but where studies have been conducted, valuable information about types and numbers of migrating insects has been obtained [8,13–15]. In Europe during the autumn, migration bottlenecks can be found within mountain ranges such as the Alps in Central Europe and the Pyrenees in Western Europe [8,15]. In these locations, mountain barriers and local winds direct insects up steep-sided valleys and through narrow mountain passes. In Central European migration bottlenecks, insects have been monitored at Randecker Maar research station in the Schwäbische Alb uplands in Germany since 1978, and over the Col de Bretolet mountain pass in the Swiss–French Alps for 12 years between 1962 to 1973 [8,16].

By contrast, migration bottlenecks along the Western European flyway have been poorly studied for insects despite the early recognition of this flyways [13,15,17,18]. The most significant of these passes is Puerto de Bujaruelo (Port de Gavarnie) on the France–Spain border in the Haute-Pyrenees, where David and Elizabeth Lack ‘chanced upon a spectacle’ of insect and bird migration on a single day in mid-October 1950 and recorded their observations [15, p. 63]. This classic study revealed impressive movements of butterflies, dragonflies and huge numbers of hoverflies, describing these as ‘the most remarkable migrants of all’ owing to their sheer abundance. Between 1951 and 1952, several other researchers briefly visited the pass, and elsewhere in the Pyrenees, and noted evidence of migration of Lepidoptera, Diptera and Odonata [17–19]. For example, in 1953, Williams *et al.* [13] studied insect migration at three sites in the Pyrenees, including the Bujaruelo Pass from 18 September to 15 October, making it the longest continuous observational study of insect migrants through a Western European migration bottleneck. They confirmed previous observations and added Hymenoptera to the list of migratory taxa, as well as recording ad lib meteorological conditions leading to the conclusions that insects do not migrate on cold, wet and sunless days [13]. While these studies made important contributions to the field, they lacked a systematic and sustained approach to the recording of insect migration, leaving the question as to the diversity of migrants using this route, the number and variation in individuals, and how this variation is affected by environmental factors. To rectify this gap in our knowledge, and to assess the current state of insect migration through the pass, we systematically quantified daytime insect migration through the Bujaruelo Pass during the September to October migration period of 2018 to 2021 inclusive.

## 2. Material and methods

### (a) Location

**Figure 1.**
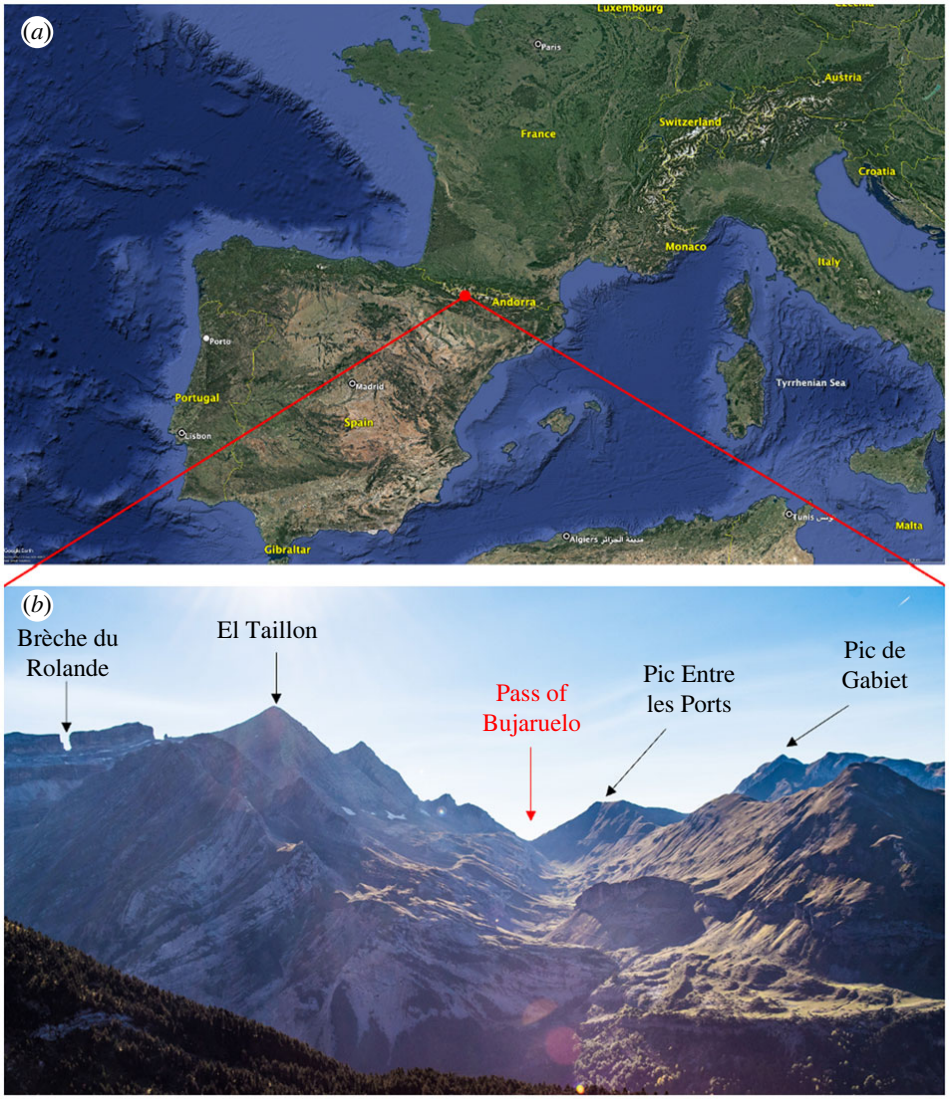
The Pyrenean mountain range and the Pass of Bujaruelo. (*a*) The fieldwork location (red marker) within the Pyrenean mountain range; image taken from Google Maps. (*b*) The valley that channels the insects over the Pass of Bujaruelo (red arrow) and the surrounding geological formations.

The Pyrenees mountain range runs along the border between southern France and northeast Spain (figure 1*a*). Within the Hautes-Pyrénées region of southwest France, the Vallée des Gaves runs north to south from the town of Argelès-Gazost before ending abruptly at the high-walled Cirque du Gavarnie (figure 1*b*). A smaller valley runs northeast to southwest from the Cirque du Gavarnie, culminating in the mountain pass of Bujaruelo where we carried out our observations (42°42'14.2″ N 0°03'51.4″ W) (figure 1*b*). The pass itself sits at 2273 m and is 30 m wide, hemmed in by high mountain peaks (Pic Entre les Ports—2476 m and Pic de Gabiet—2716 m to the north, and El Taillon—3144 m to the south; figure 1*b*). The northeast part of the pass falls within the Parc National des Pyrénées and the area consists predominately of rocky shale with patches of grass kept short through weather and grazing pressures. On the slopes leading up to each side of the pass, flowering plants grow, with Pyrenean thistle (*Carduus carlinoides*) being the only nectar source in substantial numbers. All fieldwork occurred between the months of September and October annually during 2018 to 2021. Permission to conduct observations was obtained from the Parc National des Pyrénées (France, authorization numbers: 2018-9, 2019-67, 2020-146 and 2021-33) and the Gobierno de Aragón (Spain, authorization numbers: 500201/24/2018/06141, 500201/24/2019/02174, 500201/24/2020/01724 and 500201/24/2021/01722).

### (b) Insect identification

Identification of migrating butterflies and dragonflies was carried out by eye as they flew over the pass. Smaller insects were caught with a bidirectional malaise trap continuously set up on the pass, with openings to the southwest and northeast (ez-Migration Trap, Bugdorm). Samples from this trap were collected each evening and identified to at least family level under a Leica MZ6 dissecting microscope. These identifications were used to estimate the ratios of the numbers of migratory insects recorded by the video camera. During the study periods of 2020 and 2021, the sex ratios of three Dipteran species (the hoverflies *Melanostoma mellinum* and *Eupeodes corollae*, and the muscid fly *Musca autumnalis*) were also recorded.

### (c) Determining migrants from the bidirectional malaise trap

To be included in the analysis, insect taxonomic groups had to be recorded ≥100 times in the bidirectional malaise trap counts. Migrants were determined based on a ‘southward score’, which is the percentage caught heading south in a wind category (headwind or tailwind) minus the percentage caught heading north in the same wind category:

headwind southward score=percentage  heading  south−percentage heading north,

tailwind southward score =percentage  heading south−percentage heading north.

Taxonomic groups were then sorted into three categories: ‘High-altitude’ migrants, ‘Flight Boundary Layer’ migrants (hereafter FBL migrants) and ‘Non-migratory’ based on the southward scores under different wind conditions. ‘High-altitude’ migrants includes taxonomic groups with a southward score of ≥90 in headwind conditions but with fewer than 100 individuals caught in tailwinds, presumably as they can use these favourable tailwinds to fly over the highest peaks and therefore do not use the pass. FBL migrants include taxonomic groups with a southward score of ≤100 in headwind conditions *and* ≥50 in tailwind conditions, indicating that they still use the pass in tailwinds.

### (d) Quantifying the bioflow of the larger migrants

The movements of the migrant butterflies and dragonflies were quantified by counting numbers as they passed through the 30 m wide pass during a 15 min period, once every 2 h from 10.00 to 16.00. This timing encapsulates the warmest part of the day, before and after which the diurnal insects are very unlikely to be migrating. These numbers were then scaled for the remaining 2 h between the surveys up until 18.00 by multiplying the counts of insects (*N*_1_) by 8 to reach 2 h:

$$\mathrm{number}\;\mathrm{of}\;\mathrm{insects}\;\mathrm{per}\;2\;h=8N_{1}.$$

The migratory traffic rate (MTR: number of individuals per metre per minute) of the insects was calculated by dividing the number of insects recorded in a count (*N*_1_) by the number of minutes recorded (15 min = *t*_rec_), before dividing this figure by the width of the pass (30 m = *W*):

$$\mathrm{MTR}=\frac{N_{1}/t_{\mathrm{rec}}}{W}.$$

### (e) Quantifying the bioflow of smaller migrants

Numbers of the smaller migrants were quantified from video recordings 1 min in length, taken every 15 min from 09.00 to 17.00 each day, over a 2 m wide section of the pass, as in Hawkes *et al*. [14]. The video recording monitored an area up to 2 m in height. To reach the pass, the insects must migrate up a steep valley channel before cresting a lip into the pass. The video trap was situated just after this lip. This geographical influence, coupled with the fact that the insects were often battling a headwind, meant that all insects were flying close to the ground in the flight boundary layer (approx. 1 m) as they passed the video trap. We therefore believe that no insects were missed by only recording up to 2 m in height. We scaled these raw insect counts to get a more representative estimation of the insects migrating through the Bujaruelo Pass (*R*_1_). To scale for time, raw counts of 1 min in length (*N*_2_) were multiplied by 15 to obtain representative counts for 15 min. To scale for the width of the pass (30 m) the counts were multiplied by 15 to encompass the entire width:

$$R_{1}=(N_{2}\times 15\;\min)\times 15\;m.$$

To allow a comparison between our data and other previously investigated migratory bottlenecks we calculated the migratory traffic rate, given as the number of small insects per metre per minute (m^−1^ min^−1^). The migratory traffic rate (MTR) of the smaller insects was calculated by dividing the number of insects recorded in a count (*N*_2_) by the number of minutes recorded (1 min = *t*), before dividing this figure by the width over which the counts was performed (2 m = *W*).

$$\mathrm{MTR}=\frac{N_{2}/t}{W}.$$

The video trap was unable to resolve insects smaller than 8–10 mm in length, as was noted during previous research [14]. Of the insects caught in the intercept traps that displayed migratory behaviour, 57% were too small to be resolved on the video camera. To account for this, all mentions of insect counts below include the missed insects.

### (f) Diurnal insect timings

To determine the timings at which insects fly during the day, ‘mass migration’ days were identified. To identify these days, we ordered all the days from the four years of study by number of insects recorded from highest to lowest. We then selected all migration occasions as ‘mass migration’ events until they cumulatively reached 95% of the total number of individual insects recorded; 38 days in total were identified. During 2018, the data from the video trap were not granular enough for the desired analysis, so mass migration events during 2018 are omitted from this section. Therefore, 22 separate mass migration days from the 2019, 2020 and 2021 field seasons were chosen to analyse. The mean counts obtained by the video camera from every 15 min during these days (from 09.00 to 17.00) were then calculated to identify the daily temporal distributions of migrating insects.

### (g) Meteorological data

Weather conditions were taken from MeteoBlue [20] because of the increased consistency of those recordings compared with our *in situ* recordings, and the higher fidelity of ‘difficult to record’ variables such as precipitation or sunshine. The meteorological variables recorded were: average daily temperature (°C), wind heading, total daily precipitation (mm), windspeed (m s^−1^), and daily sunshine amount (min). Some of our data points were missed owing to inclement weather on the pass causing weather data collection to be unfeasible, although the traps were still collecting. Additionally, the MeteoBlue recordings covered a greater geographical area, which is relevant for the insects that would have begun their journeys at locations away from the pass. Our *in situ* recordings of temperature closely matched the patterns in the MeteoBlue data (electronic supplementary material, figure S1). Large-scale wind patterns were visualized using the Web application Ventusky [21].

### (h) Analysis of meteorological factors

Regression analysis was undertaken to determine how environmental factors affected the numbers of insects migrating through the pass. Count of the insects (integrating numbers from the camera trap and the larger insect counts) was the response variable, and the explanatory variables that were investigated were: (1) degree of headwind, (2) total daily precipitation, (3) minutes of sunshine per day, (4) average daily temperature, (5) windspeed along with interactions between (6) headwind and windspeed (to investigate if windspeed has a positive or inhibitory effect depending on wind direction), (7) sunshine and temperature (to investigate if there is a multiplicative effect) and (8) sunshine and precipitation (to investigate if days with sunny and rainy intervals can still have high migration). The variable 'minutes of sunshine per day' was normalized to a scale of 0–1 and the degree of headwind was adjusted to a scale from −1 (for a full tailwind) to 1 (for a full headwind); see electronic supplementary material, equation S1. The variables were also ranked by importance [22] by averaging their Akaike information criteria (AICs) over 15 models in which they appeared in every combination of 3 or 4 variables, including their interactions. All analyses were conducted using R version 4.2.1 and RStudio version 2023.03.0. [23]. The packages DHARMa [24] and lmtest [25] (which uses generalized linear models [26]) were used in model verification to determine that insect count data were both over-dispersed and zero-inflated. Zero-inflated negative-binomial regressions from the package pscl [27] were subsequently used to model the zero-heavy insect count data. The cauchit link function was found to provide the best model fit of the zero-inflation model. An all-subsets approach using the package MuMIn [28] for variable selection by AIC was used to determine which variables improved the model fit, with variables treated separately for the zero and count portions of the model. Confidence intervals of model predictions were computed using a bootstrap obtained from Zeileis *et al*. [29].

The full R code analysis has been made available in the electronic supplementary material.

### (i) Fine temporal scale effect of sunshine on insect migration

Days of mass migration events were chosen for sunlight analyses as all the other environmental conditions affecting insect migration were likely to be similar across these events. Sunlight analysis was performed at a fine temporal scale on the mountain pass using the video trap footage from the 2021 field season to check for presence/absence of sunlight. Each of 382 occasions was classified as either sun present or sun absent and the number of insects within each measurement occasion was recorded. For the larger insects recorded in the bi-hourly counts, the amount of sunlight per count was recorded on a sliding percentage scale (0–100%) rather than just the presence/absence as with the camera data (*n* = 133). Therefore, a linear model was used to assess the relationship.

## 3. Results

### (a) Assemblage of insects migrating through the Pyrenees

**Figure 2.**
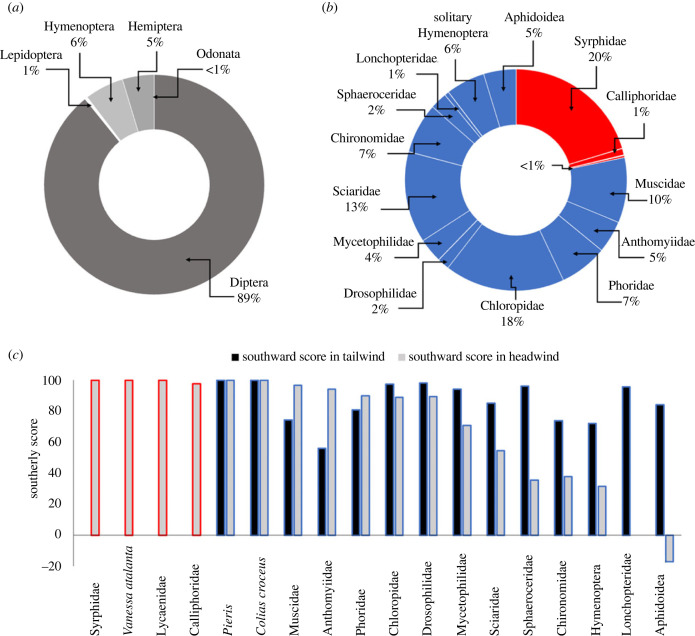
Classification of the migratory assemblage. Average ratios of insects showing migratory behaviour collected in the intercept trap and butterfly counts over 4 years sorted by (*a*) order and (*b*) family. (*c*) Migratory behaviour of insect groups based on southward scores. ‘High-altitude’ migrant insect groups (red), and ‘Flight Boundary Layer’ migrant insect groups (blue). Missing values indicate fewer than 100 individuals in the sample of head- or tailwinds. Southward scores of 100 indicate all individuals were going south, 0 that the same number were going south as north, and negative values indicate that more were going north than south under a particular wind condition.

The complete assemblage of all migratory insects consisted of 20 families from five orders (electronic supplementary material, tables S1–S3): Diptera (89% of total migratory insect number), Hymenoptera (6%), Hemiptera (5%), Lepidoptera (1%) and Odonata (less than 1%) (figure 2*a*). From the southward scores, determined from patterns of movement under different wind conditions, four insect types were classed as ‘High-altitude’ migrants: hoverflies (Syrphidae), red admiral butterflies (*Vanessa atalanta*), blue butterflies (Lycaenidae), and blowflies (Calliphoridae) (figure 2*b,c*; electronic supplementary material, table S2); 12 insect types were classed as ‘FBL’ migrants: cabbage white butterflies (*Pieris* spp.), clouded yellow butterflies (*Colias croceus*), certain Diptera families (Muscidae, Anthomyiidae, Phoridae, Chloropidae, Mycetophilidae, Sciaridae, Lonchopteridae and Sphaeroceridae), solitary Hymenoptera and aphids (Aphidoidea) (figure 2*b,c*; electronic supplementary material, table S3). Further species and groups, like the painted lady butterflies (*Vanessa cardui*)*,* hummingbird hawkmoths (*Macroglossum stellatarum*) and some dragonflies (e.g. *Aeshna mixta* and *Sympetrum striolatum*), were seen performing directed movements southwards over the pass, but their numbers did not exceed the 100-individual threshold to be included in further analyses. It is thought that the numbers recorded of dragonflies and hummingbird hawkmoths are likely an underestimation, because their darker coloration and fast flight made them difficult to count. The primary ecological roles (occasionally insects had two or more primary roles) of the migratory assemblage were identified from the literature and this revealed that the most numerous were pollinators (87.5% of total insects counted from both video camera and butterfly counts). Other, sometimes overlapping roles, included decomposers (33.6%), pest predators (22.2%) and pests (5%), with 100% of the total insect assemblage contributing to nutrient transfer (electronic supplementary material, table S4).

### (b) Quantifying insect bioflows through the Pass of Bujaruelo

**Figure 3.**
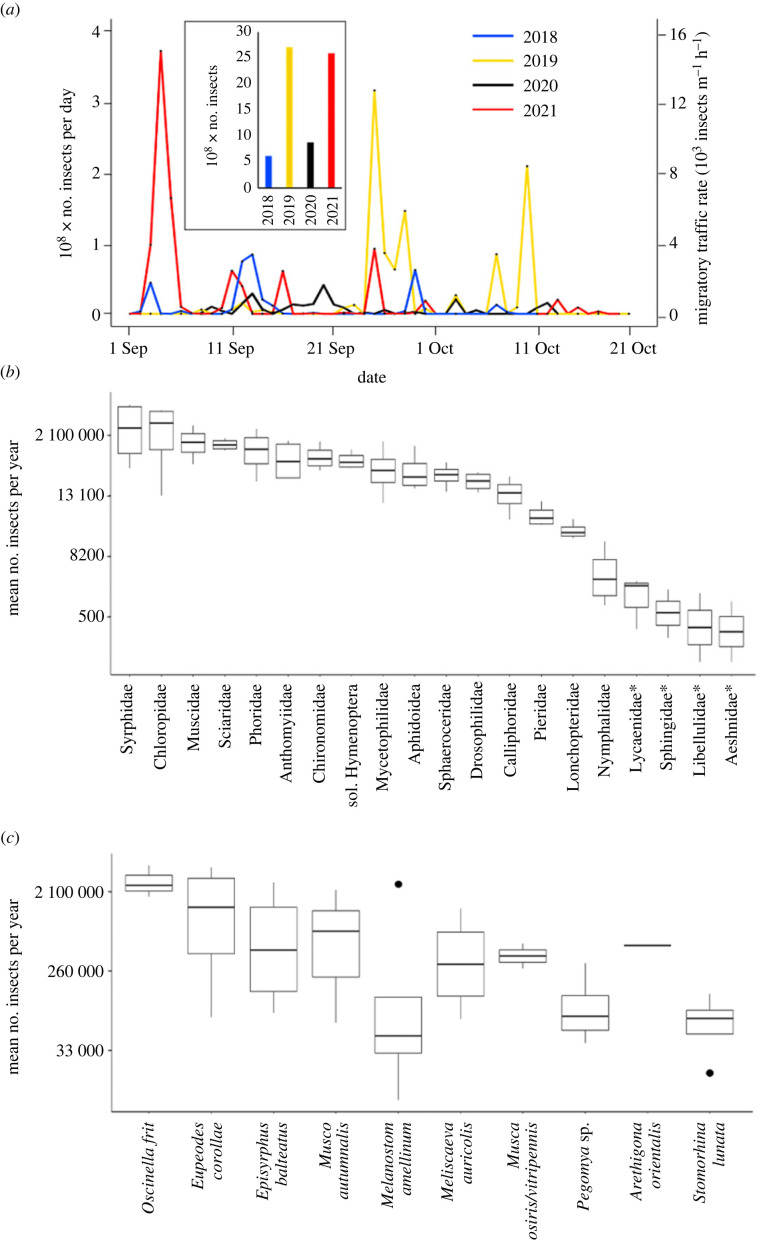
Migratory insect bioflows. (*a*) Number of insects and migratory traffic rates per day across four years based on video trap data (not including the estimated missed insects or butterflies—see Methods). (*b*,*c*) Box and whisker plots detailing the variation in insect families (*b*) and the top ten most numerous species (*c*) across each season.

In total, scaled counts from malaise traps, video traps and butterfly counts reveal an average of over 17.1 million insects traverse the Pass of Bujaruelo each autumn. However, variation from year to year ranged from 6.2 million insects to 27.1 million insects, i.e. an annual variation of 4.4-fold (figure 3*a* inset) (a full breakdown can be found in electronic supplementary material, table S5). The majority of these were the smaller insects recorded by the video traps, which made up 99.6% of total migrants or 68 million individuals over the four years. Numbers through the pass varied between 0 and 3683 individuals per metre per minute (m^−1^ min^−1^) across the four years of data collection. The temporal distribution of all insects recorded by the camera trap over a day (09.00–17.00) reveals the peak of insect numbers occurs at 14.45 (electronic supplementary material, figure S2), about 1 h after the solar noon.

Butterflies composed only 0.4% of the total, with just over 260 000 individuals across the four years, with annual counts varying from 38 500 to 107 000. Butterfly migratory traffic rates varied between 0 and 4 individuals m^−1^ min^−1^ across the four years. Of the butterflies, the most abundant family were the Pieridae (*C. croceus* and *Pieris* spp. at 40 000 and 15 000 individuals per season respectively) while the common darter dragonfly (*S. striolatum*) was the most abundant dragonfly at 400 per season.

Of the migrant Diptera, Syrphidae were the most abundant, averaging 3.1 million per season and making up 20% of the migratory assemblage (figure 3*b*). Chloropidae, Sciaridae, Muscidae and Phoridae all averaged over a million individuals each season, with lower numbers seen for the Anthomyiidae, Mycetophilidae, Drosophilidae and Calliphoridae. At the species level, the most abundant in the bidirectional malaise traps were the *Oscinella frit* grass flies (Chloropidae) and the hoverflies *Eupeodes* *corollae* and *Episyrphus balteatus* (Syrphidae), while flies from the Muscidae (such as *Musca autumnalis*) and from the Anthomyiidae (such as *Pegomya* sp.) were also abundant (figure 3*c*). Larger dipteran migrants (*Eristalis tenax* and *Scaeva* spp.) were, in general, able to avoid the malaise trap despite being present in substantial numbers in the video traps. In general, sex ratios were skewed toward females for *Melanostoma mellinum* (F 65%, M 35%, *n* = 826), *E. corollae* (F 96%, M 4%, *n* = 929) and *Mu. autumnalis* (F 72%, M 28% *n* = 1148). In total, all migratory taxa constituted an average of 140 kg of biomass each season.

### (c) The effect of environmental factors on the number of insects migrating

Over the 191 observed days, the amount of insect migration observed daily varied hugely from 0 to 3.7 million individuals. We investigated the influence of wind direction, daily rainfall, minutes of sunshine, temperature and windspeed on the numbers of insects migrating through the pass. As many combinations of weather conditions resulted in days with zero observed insect migration, we used a zero-inflated regression model to separately quantify which variables affected the chance of observing migration, and the amount of migration observed if it did occur.

**Figure 4.**
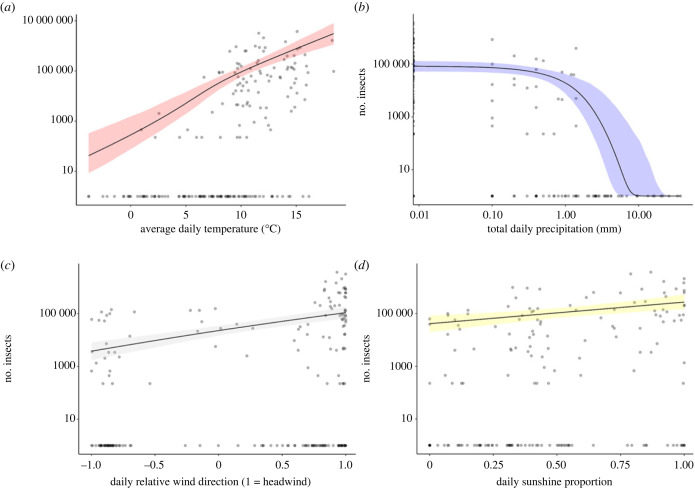
The relationship between the number of insects migrating through the Pass of Bujaruelo and meteorological variables (*a*) average daily temperature, (*b*) total daily precipitation, (*c*) average daily relative wind direction, and (*d*) daily sunshine proportion. Model fits were plotted using the median values for other predictor variables, with 95% confidence intervals.

Temperature was the most influential predictor (see electronic supplementary material, table S6) and had a consistently positive effect on both the chance of observing insect migration as well as the number of insects counted (figure 4*a*). Daily rainfall strongly inhibited migration, with migration occurring on only on 26/84 rainy days, compared with migration occurring on 70/107 dry days, and no migration was observed on days with over 1.4 mm of rainfall (figure 4*b*).

Wind direction was also a strong predictor of migration, with a higher chance of observing insects on days with a headwind and a positive influence on the number of insects counted on days in which migration occurred (figure 4*c*). Neither windspeed nor the interaction between windspeed and the degree of headwind had a noticeable effect on either the chance of observing insect migration or the quantity of insect migration. To investigate the role of wind over a broader scale we visualized wind patterns using the Web application Ventusky over Western Europe, centred on the Pyrenees. On 31 out of 38 mass migration days, there was a tailwind or low windspeeds local to the northern (French) side of the mountains that met a headwind coming up from the south (Spain) along the ridgeline of peaks (electronic supplementary material, figure S3).

The number of minutes of sunshine did not significantly affect the chance of observing migration; it had a strong effect on the number of insects counted, with full sunlight increasing the number of observed insects fourfold compared with a completely overcast day (figure 4*d*), although this increase was not additive with temperature when predicting the quantity of migration. To account for fine-scale variation in the effect of direct sunshine on migrant numbers we used 15 min counting periods from the migration camera trap data. We found that the amount of sunshine significantly affected the numbers of small insects recorded moving through the pass by the migration camera. Each 15 min counting period when it was sunny contained 16-fold more insects on average than when the sun was hidden (*t* = −6.8, *p* ≤ 0.0005). For the larger insects, a linear model was used to show a significant positive relationship between the larger insects and sunlight (*R*^2^ = 0.06, *F*_1,130_ = 8.347, *p* ≤ 0.005). For full parameter estimates, see table 1. Model details including ΔAIC analysis can be found in electronic supplementary material, table S7. A breakdown of the variable importance from a round-robin analysis of different combinations can be found in electronic supplementary material, table S6.

**Table 1.** The parameter estimates for the effect of environmental factors on the number of insects migrating. A zero-inflated negative binomial model of insect migration counts was used. This would predict number of insects observed on days in which migration occurs in the count model, and the probability of parameters resulting in a day of zero migration (note that positive values indicate a contribution towards the chance of zero migration). The *Z*-value is a measure of how many standard deviations an estimate is from the null hypothesis. The intercept gives a value to add to everything else when making predictions (it would be the number of insects when there is 0 net headwind, 0 mm rain, 0 sunshine and 0°C temperature).

parameter	estimate	standard error	*Z*-value	*p*-value
count model; estimates on log scale				
intercept	4.29	1.10	3.91	<0.001
headwind (−1 to 1)	1.36	0.19	7.09	<0.001
daily rainfall (mm)	−1.26	0.45	−2.81	0.005
daily sunshine (0–1)	6.15	1.86	3.30	<0.001
average temperature (°C)	0.56	0.10	5.54	<0.001
sunshine : temperature	−0.43	0.16	−2.73	0.006
zero model; estimates on cauchit scale				
intercept	3.02	1.15	2.61	0.008
average temperature (°C)	−0.35	0.11	−3.07	0.002
daily rainfall (mm)	0.67	0.33	2.01	0.044
headwind (−1 to 1)	−0.73	0.28	−2.58	0.010

## 4. Discussion

More than 70 years after it was first discovered as an important insect migration bottleneck [15], we returned to the Pass of Bujaruelo to systematically quantify the flow of day-flying insects migrating through the pass and to gain an understanding of the scale of the entire diurnal migration along the Western European insect flyway. We estimate that during our observation period of September to October 2018–2021, an average of 17.1 million insects traversed the pass each season heading south on their migratory journeys. Their migrations continue south into Spain and, for at least some, into Africa, as evidenced by records of migratory Syrphidae in Morocco during the autumn, and the suitability of overwintering sites in the Sahel region of Sub-Saharan Africa for migratory butterflies [30,31]. In the following sections we discuss meteorological conditions that predict migration events, the diverse range of species involved, the scale of the Western European flyway and the ecological impact of the individual day-flying insects crossing the Pyrenean mountain range each autumn.

### (a) Comparisons with historical observations at mountain passes

A lack of systematic data collection performed in the Pass of Bujaruelo in the 1950s makes comparisons challenging, particularly for Diptera, as migration rates through the pass were only compared with other migrants: e.g. ‘at least twenty times, and perhaps a hundred times, as numerous as the dragonflies', which themselves were moving at a rate of ‘at least several thousand an hour’ [15]. By contrast, studies focusing on hoverflies in the 1960s and 1970s in the Alps utilized a similar, but larger, intercept trap to ours (2 m^2^ versus 16 m^2^) [32], making comparisons more straight-forward following the eight times scaling of our numbers. The most common hoverfly in our study, *Eupeodes corollae*, with an average (over the four years) scaled count of 6100 was just over 46% of the average of 13 228 individuals caught in the 12 years between 1962 and 1973 in the Alps [8]. By contrast, the most common hoverfly in the Alps study was *Episyrphus balteatus*, with our counts reaching only 2.6% of the 107 602 individuals caught by Aubert *et al.* [8]. Comparing numbers from many years apart, from different traps and from different sites is problematic and we do not know how contemporary numbers would compare across sites. However, work at Ran Decker Maar research station in the mountains of southwest Germany found precipitous declines of 97% since 1970 for aphidophagous migratory hoverflies [16], suggesting declines at our fieldsite in the Pyrenees could be similar [16]. If these declines have occurred consistently across Europe, the numbers moving through the Pass of Bujaruelo during the study period of the early papers (1950s) may have been many times more than what is currently observed. Of the other insect groups, we occasionally saw butterflies passing through in numbers exceeding the rate of 500 h^−1^ noted by Lack & Lack [15], but while huge numbers of dragonflies were twice mentioned in the 1950s papers, for example ‘an uncountable stream of [*Sympetrum striolatum* dragonflies]’ [17, p. 3], during our four years at the Pass of Bujaruelo, we rarely saw dragonflies passing in such numbers.

We found strong evidence of migratory behaviour in small species not noted during the 1950s studies, likely owing to a lack of systematic sampling techniques. The Chloropidae, Phoridae, Drosophilidae, Mycetophilidae, Sciaridae, Sphaeroceridae, Chironomidae, Lonchopteridae, solitary Hymenoptera and the Aphidoidea all showed migratory behaviour. Previous studies have regarded these insects as being simply at the mercy of air currents without the ability to choose their own direction [11]. However, the consistent appearance of these insects in the southward-heading side of our bidirectional malaise trap despite headwinds suggests that these smaller insects are capable of directed migration. Further studies are needed to ascertain how these smaller species can select favourable conditions for movement, the distances they may be travelling, and if they utilize similar compasses to larger Dipteran migrants [33]. Finally, on a few occasions, migration of interesting species was noted, but as they occurred in small numbers they fall outside of our criteria and records of these species were made on an ad hoc basis. Details of these migrants can be found in the electronic supplementary material, results and discussion.

### (b) The Western European migratory assemblage and their ecological roles

Pyrenean migration bottlenecks have been relatively poorly studied for insects despite the early recognition of these flyways [13,15]. We identified 20 families of day-flying insects from five orders showing migratory behaviour as judged by directional movement through the Pass of Bujaruelo.

For three migratory Dipteran species (*Melanostoma mellinum, E. corollae* and *Musca autumnalis*), the sex ratios were recorded. In all cases, females were the more numerous migrants (65% *M. mellinum*; 96% *E. corollae*; 72% *Mu. autumnalis*). This finding contrasts with springtime observations in Cyprus for *M. mellinum* where the sex ratios were balanced (male 49 : 51 female) (W. L. Hawkes 2019, unpublished observation). Autumnal studies in Latvia showed that migratory *Eristalis tenax* hoverflies were 67% male-weighted (W. L. Hawkes, unpublished observation), while studies also during the autumn in the Czech Republic revealed the sex ratios of *M. mellinum* were approximately 50 : 50, and *E. corollae* was more male-weighted [34]. In summary, our study of a select group of Diptera more closely resembles the sex ratios obtained in the Alps, where females are the more common sex during autumn (70% *M. mellinum*; 79% *E. corollae* [35]). Migratory hoverflies are thought to mate and store sperm before beginning their autumnal migration and undergo diapause during their long journeys. This makes the female flies key to continue their populations at lower latitudes. Sex ratios recorded at higher latitudes suggest that males begin the migratory journey, but perhaps owing to their poorer migratory ability, they die along the way, leaving females to overwinter.

**Figure 5.**
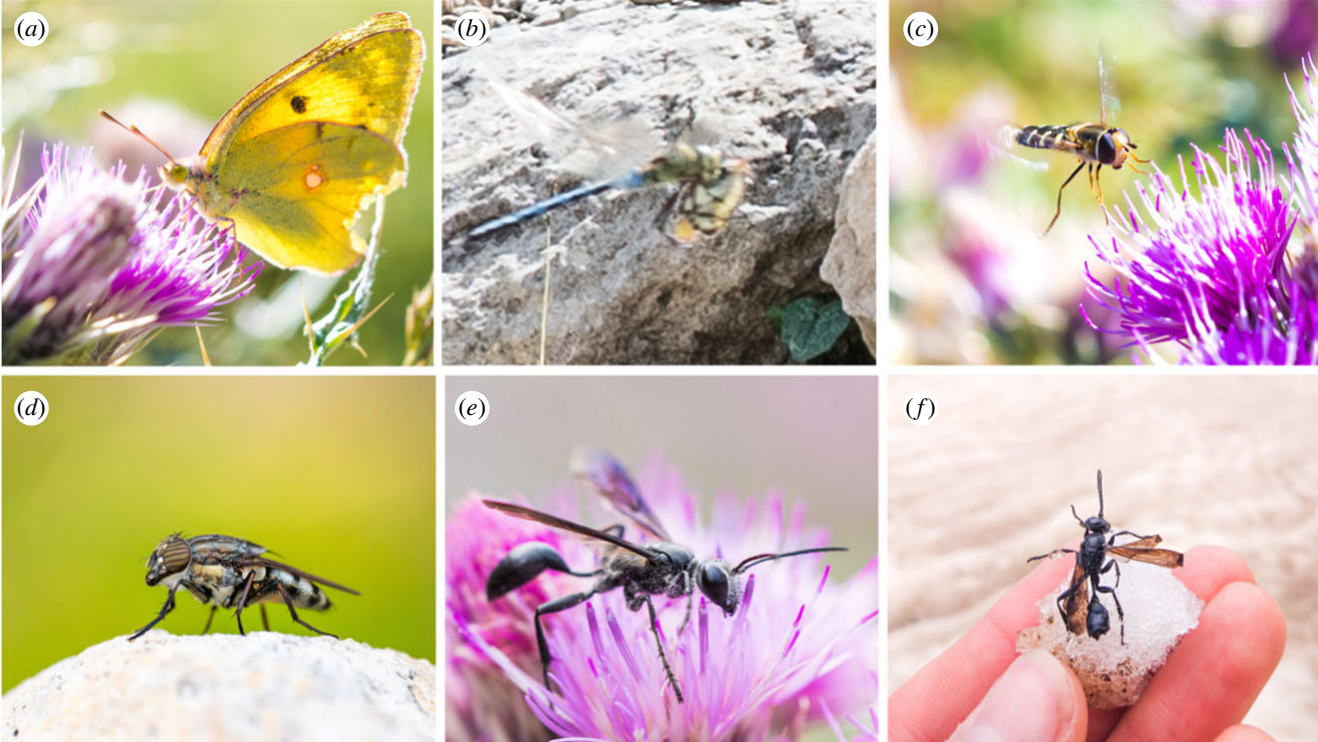
Some migratory insects of the Pyrenees. (*a*) Clouded yellow (*Colias croceus*), (*b*) migrant hawker (*Aeshna mixta*) eating a clouded yellow (*Colias croceus*), (*c*) pied hoverfly (*Scaeva pyrastri*), (*d*) locust blowfly (*Stomorhina lunata*), (*e*) *Isodontia mexicana* feeding on Pyrenean thistle (*Carduus carlinoides*) within the Puerto de Bujaruelo pass, (*f*) *Isodontia mexicana* found dead on a glacier at approximately 2750 m above sea level.

The ecological roles of the migrants were dominated by pollinators, which made up nearly 90% of the insects recorded, followed by pest species (36%), decomposers (34%), and natural enemies of pests (20%), while all contributed to the transport of nutrients (100%). A full discussion of these roles is presented in the electronic supplementary material, results and discussion. The spread of these ecological roles over large geographical areas, and through time, is likely to have major consequences for ecosystem function. For example, migratory pollinators can link geographically isolated plant populations through their movements, allowing for gene flow between these populations [36]. The movement of pest species may lead to direct damage to crops while also increasing rates of gene flow, and thereby increasing the chance of resistance to pesticides arising and spreading [37]. In addition, the movement of large amounts of biomass through the pass, estimated at a little over 140 kg and equating to 14 kg of nitrogen and 1.4 kg of phosphorus, forms part of a larger movement of insects which together represents the largest terrestrial bioflow [2], and has major potential benefits, including revitalizing various ecosystems along with the arrival of migratory insects through adding nutrients to soils (figure 5).

### (c) Environmental factors influencing migratory intensity and total numbers

Environmental factors have been shown previously to affect the numbers of insects arriving at migratory bottlenecks [14]. Our model from the Pyrenees showed that, listed in order of explaining the most variation in insect numbers, the average daily temperature, total daily precipitation, degree of headwind and number of minutes of sunshine were significant in affecting the number of insects migrating through the pass.

Temperature was found to be positively correlated with insect number, a finding that is consistent with findings of migrating dragonflies in Latvia, hoverflies in North America, and migratory insects of all taxa in Cyprus [6,14,38]. Daily precipitation, in contrast, was a strong inhibitor of migration, with no migration at all observed on days when there was more than 1.4 mm of rainfall. These findings suggest that insects can migrate in mild rainy conditions, as corroborated by radar data where the researchers found that mild and moderate rainfall diminished migratory insect numbers but did not cause full termination of flight [39].

The degree of headwind was also a strong predictor of whether insect migration occurred. The topography of the Pyrenees means that on days when a tailwind is present, the insects can ride updrafts over the higher than 3000 m peaks, and we observed migration of flies and butterflies through the Brèche de Roland (2804 m) and over the peak of Taillon (3144 m) on multiple occasions. On headwind days, however, insects are forced down low into their ‘flight boundary layer’ [40] to avoid the winds and are guided by topographical features through mountain passes like Bujaruelo. Therefore, in contrast to studies in Latvia and Cyprus (among others) [6,14], ‘high-altitude’ migrant insects were recorded almost exclusively during headwinds (figure 4*a*). Insects are well known to choose favourable tailwinds to aid their migratory journeys [2,41–45], but our observations show that they are also capable of migrating in headwind conditions if they must. The most likely explanation is that of orographic wind, the process by which a mass of air is lifted by a large geographical feature such as a mountain range, creating winds that blow towards the mountain tops [46]. These local wind conditions are therefore as important as the large synoptic-scale tailwinds blowing from France into Spain, with regards to the insects migrating through the Pyrenees. Therefore, while insects can utilize a tailwind to help power their flight south, on reaching the ridgeline they may need to counteract a headwind to continue their journey.

The total daily sunlight's positive correlation with insect number is also consistent with previous Pyrenean observations by Williams *et al*. [13, p. 402], who stated that ‘sunshine is a major factor determining […] migration’, and findings that the effects of sunlight and temperature on insect migration are tightly linked [6,47]. When analysing the effect of sunlight on insect numbers at a fine scale, we found that insects were 16-fold more abundant than when there was no sun. Insects are often highly sensitive to changes in temperature due to the presence of sunlight as their small body size is particularly susceptible to heat loss, and they may also require the sun to orientate [33,48,49].

In summary, predictors of large migration events over the pass are as follows: a warmer temperature, no rain, the presence of a headwind, and the presence of sunshine. This predictive model can be applied to other passes in high mountain ranges (electronic supplementary material, figure S4).

### (d) Total numbers traversing the Pyrenees and the Western European insect flyway

Numerous studies have found a consistent south or southwest bias in migratory insect headings across Europe [42,44,45,50], suggesting insects from a large geographical area are filtered down into the Iberian Peninsula, passing through the Pyrenees each season. This makes insect migration bottlenecks within the Pyrenees important locations for censusing species and monitoring numbers. We estimate that the total number of insects moving across the Pyrenees mountain range reaches into the tens of billions. This number is of comparable size to those from radar studies across a similar-sized area [2]. Insects are known to cross the Pyrenees not only in the centre where the Pass of Bujaruelo is situated, but also along the coasts. Williams *et al*. [13] observed some southward movement of butterflies in October along the coast at Argelès-sur-Mer, where the Pyrenees descend to the Mediterranean Sea. Similar southward movements occur along the Atlantic seaboard [51,52]. To accurately quantify the total number of migratory insects crossing the Pyrenees, extensive deployment of monitoring resources and techniques is needed, including the use of vertical-looking radars [2,53].

## 5. Conclusion

Our four-year study of insects migrating through a Pyrenean mountain pass has revealed a remarkable diversity and abundance of migratory insect taxa. Additionally, based on the south–southwest bias of insect movement during the autumn season in Europe, the insects migrating through the Pyrenees are likely to have originated from across a large area of Western Europe [44,50]. More long-term studies of migratory bottlenecks are required to expand the monitoring of insect populations and to avoid ‘shifting baseline syndrome’ [54]. We suggest studies incorporate use of low-cost migration cameras to collect data during headwinds and, where possible, entomological radar to fully quantify the migration in tailwind conditions, though we recognize the potential difficulty in locating and maintaining such technology in remote locations. Our study has shown that many more insect taxa show directed migratory behaviour than we were previously aware of and that research into the ecological benefits of these insect migrants is of importance, especially to the agricultural and horticultural sectors. These findings highlight the benefits accrued from insect migration (including nutrient transfer, pest control, pollination and decomposition) and provide key predictors of migratory events, vital in understanding population changes in the era of anthropogenically induced environmental change.

## Data Availability

Data are available as electronic supplementary material and in Dryad at https://doi.org/doi:10.5061/dryad.vq83bk40v [55]. Supplementary material is available online [56].
